# Methodological Bias Can Lead the Cochrane Collaboration to Irrelevance in Public Health Decision-Making

**DOI:** 10.1371/journal.pntd.0004165

**Published:** 2015-10-22

**Authors:** Antonio Montresor, David Addiss, Marco Albonico, Said Mohammed Ali, Steven K. Ault, Albis-Francesco Gabrielli, Amadou Garba, Elkhan Gasimov, Theresa Gyorkos, Mohamed Ahmed Jamsheed, Bruno Levecke, Pamela Mbabazi, Denise Mupfasoni, Lorenzo Savioli, Jozef Vercruysse, Aya Yajima

**Affiliations:** 1 Control of Neglected Tropical Diseases, World Health Organization, Geneva, Switzerland; 2 Children Without Worms, Task Force for Global Health, Decatur, Georgia, United States of America; 3 WHO Collaborating Centre for Neglected Tropical Diseases, Public Health Laboratory Ivo de Carneri, Zanzibar, United Republic of Tanzania; 4 Neglected Infectious Diseases, Pan American Health Organization, World Health Regional Office for the Americas, Washington, DC, United States of America; 5 Neglected Tropical Diseases, World Health Organization Regional Office for the Eastern Mediterranean, Cairo, Egypt; 6 Malaria, Other Vectorborne and Parasitic Diseases, World Health Organization Regional Office for Europe, Copenhagen, Denmark; 7 WHO Collaborating Centre for Research and Training in Parasite Epidemiology and Control, Department of Epidemiology, Biostatistics and Occupational Health, McGill University, Montreal, Quebec, Canada; 8 Vector-Borne and Neglected Tropical Diseases Control, World Health Organization Regional Office for South-East Asia, New Delhi, India; 9 WHO Collaborating Centre for the Monitoring of Anthelminthic Drug Efficacy for Soil-Transmitted Helminthiasis, Department of Virology, Parasitology and Immunology, University of Ghent, Ghent, Belgium; 10 Global Schistosomiasis Alliance and Public Health Laboratory Ivo de Carneri, Zanzibar, United Republic of Tanzania; 11 Malaria, Other Vectorborne and Parasitic Diseases, World Health Organization, Regional Office for Western Pacific, Manila, Philippines; University of Queensland, AUSTRALIA

## Aims and Limits of the Cochrane Collaboration

The Cochrane Collaboration (CC) was created in 1993 with the aim of systematically reviewing published research in order to facilitate the selection of appropriate interventions by health professionals and policy-makers [1]. CC systematic reviews focus on a wide range of health-care interventions and typically consider evidence only from randomized controlled trials (RCTs). Because they rely on chance to minimize the potential for epidemiological confounding, RCTs are commonly acknowledged as the strongest, least biased source of evidence on particular therapies or medical interventions for clinical practice. Similarly, it is well known that their utility in evaluating public health interventions is not always optimal [2] and, as a consequence, can result in distorted conclusions [3].

We specifically refer to a series of CC systematic reviews aimed at assessing the benefits of deworming for soil-transmitted helminthiases (STH) [4–6] in children, which we believe is affected by a significant methodological bias. Two essential characteristics of this intervention, and of the infections it targets, were not considered by the reviewers when they chose to restrict evidence to data derived only from RCTs.

## Recovery from STH-Associated Morbidity Is a Long-Term Process

The World Health Organization (WHO) recommends a sustained program of mass deworming for preschool-age and school-age children in areas endemic for STH, corresponding to 10–12 years of treatment for each child [7]. The aim of such a programme is to keep STH infections to as low a level of intensity as possible in order to prevent and eliminate morbidity, thus protecting a child during his or her physical and cognitive development [7]. The “intervention” to evaluate is therefore not represented by one or two rounds of treatment but by the cumulative deworming experience extending throughout childhood. The fact that RCTs have considerably shorter follow-up times means they cannot capture the real effects of the deworming intervention, and conclusions drawn from this evidence risk being severely biased.

The distribution of worms among human hosts is not uniform; only a minority of individuals in a community will have infection at a level sufficiently high to cause morbidity (i.e., at moderate or high intensities of worm burdens) [8]. Estimates indicate that, where the prevalence of infection with *Ascaris lumbricoides* is 50%, approximately 20% of the children in the community will have moderate- or high-intensity infections, and therefore exhibit morbidity [9]. However, deworming tablets are administered to the entire child population living in an endemic area (because of public health considerations such as the high cost and logistical burden of test-and-treat approaches, the low sensitivity of field-applicable diagnostic techniques, the relative safety of the medicines, the limited health infrastructure and poor access to treatment, and the low health-seeking behavior, among others). Consequently, the deworming intervention will directly benefit only a portion of the treated children, and will obviously provide no benefit to children who are not infected. It is therefore unreasonable to evaluate the benefits of deworming among all the children who are treated, instead of only among those who are infected.

Not surprisingly, the CC review concludes that the intervention may improve weight gain only in children “known to have worm infection” [6]. A systematic review is hardly necessary to point out that children without worms do not directly benefit from the administration of a deworming tablet.

In conclusion, the main challenges of using RCTs to evaluate the benefits of deworming include: (1) the short evaluation time periods relative to the longer time needed to observe accrued benefits; and (2) the need to assess the outcome of the intervention by pooling together infected and uninfected children alike, thus diluting the known benefits.

The use of RCTs or quasi-RCTs for the evaluation of deworming interventions has already received considerable criticism [7,10–15]. However, the most recent CC review on this topic [6] does not address these criticisms, but rather perseveres in its biased methodological approach, thus highlighting its considerable limitations. Not only is such a review of little value in guiding global deworming policy, it could also generate confusion among public health planners and practitioners in endemic countries, thus contributing to possible withdrawal from treatment of millions of children suffering from STH.

We are convinced of the need to properly evaluate deworming interventions which, despite their simplicity, carry significant logistic and cost burdens. The cost-benefits of such interventions need to be compared with other health interventions (such as vaccination, sanitation, and maternal and child health interventions). A proper evaluation should be organized.

**Some Examples of Studies Demonstrating the Importance of Maintaining a Low Worm Burden**

- The amount of blood lost to heavy-intensity hookworm infection has been precisely measured [16,17]. Severely infected children lose more than double the daily iron requirement [17]. It is especially difficult for those with limited iron dietary input to compensate such a daily loss of iron [17,18].
- In areas of high endemicity, women given albendazole had a lower rate of severe anemia during pregnancy [19]. Birth weight of infants of women who had received albendazole significantly improved, and infant mortality at 6 months fell dramatically [20].
- Several hospital-based studies have documented and quantified an elevated mortality due to:
  - intestinal obstruction caused by heavy intensity infections with *A*. *lumbricoides* [21] (morbidity 12 million cases; mortality 10,000 cases); and
  - dysentery syndrome caused by heavy-intensity infections with *Trichuris trichiura* [22,23].
- Evidence from veterinary studies in experimentally infected pigs has demonstrated damage to the gut surface—flattening or atrophy of villi—and the consequent maldigestion and malabsorption in moderate- and heavy-intensity ascariasis [24].
